# *Serratia marcescens* causing recurrent superficial skin infections in an immunosuppressed patient

**DOI:** 10.1002/ski2.283

**Published:** 2023-09-13

**Authors:** Sarah E. Drummond, Akash Maliampurakal, Saranaz Jamdar, Lucy Melly, Susan Holmes

**Affiliations:** ^1^ Department of Dermatology Glasgow Royal Infirmary Glasgow UK; ^2^ Department of Microbiology Glasgow Royal Infirmary Glasgow UK; ^3^ Department of Pathology Queen Elizabeth University Hospital Glasgow UK; ^4^ School of Medicine University of Glasgow Glasgow UK

## Abstract

*Serratia marcescens* is a gram‐negative bacterium found commonly in water and soil. Initially thought to be non‐pathogenic, it is now recognised as an important cause of nosocomial and opportunistic infections. Skin infections are rare, but cases of *S. marcescens* causing ulcers, abscesses and necrotizing fasciitis have been reported. We report an unusual cutaneous presentation of *S. marcescens* in an immunosuppressed patient. A 77‐year‐old man under review for non‐melanoma skin cancer in the context of a previous cardiac transplant, presented with an asymptomatic scalp eruption. Immunosuppressive medications included ciclosporin 90 mg twice daily (2.5 mg/kg/day) and mycophenolate mofetil 1 g twice daily. Physical examination revealed well‐defined annular and polycyclic patches with brownish crusting across his scalp. Bacterial culture demonstrated a heavy growth of *Staphylococcus aureus* sensitive to flucloxacillin. The patient was treated with 7 days of flucloxacillin 500 mg four times daily. Despite this, the eruption extended. Skin biopsy demonstrated epidermal spongiosis, florid dermal inflammatory cell infiltrate and abundant bacteria and neutrophils in the parakeratotic crust. Fungal stains were negative as was direct immunofluorescence. Repeat culture demonstrated heavy growth of *S. marcescens* sensitive to ciprofloxacin. The patient was treated with 10 days of oral ciprofloxacin 500 mg twice daily along with 1% hydrogen peroxide cream topically with significant clinical improvement. Microbiological review indicated that a gram‐negative organism was present in the initial scalp swab. In addition, *S. marcescens* had been detected previously on a skin swab from a recent transient eruption on the torso. Further, a heavy growth of a coliform bacillus was demonstrated in a similar eruption on the chest in 2013. It was concluded that the patient was likely colonised with *S. marcescens* which appeared to have caused recurrent superficial skin infections over several years. We report this case to highlight an unusual clinical presentation of cutaneous *S. marcescens* infection. This should be considered in the differential diagnosis of skin eruptions in immunocompromised patients. Clinical information detailing a patient’s immunosuppressed state must be supplied on microbiology requests to allow accurate interpretation of results, and consideration of organisms which may otherwise be overlooked or considered contaminants.

## Graphical abstract

We report a case of a 77 year‐old organ transplant recipient with an annular crusted eruption on his scalp. *Serratia marcescens* was detected on bacterial culture and the eruption improved significantly with oral antibiotics. *S. marcescens* is a rare cause of skin infection and should be considered in the differential diagnosis of skin eruptions in immunosuppressed patients.

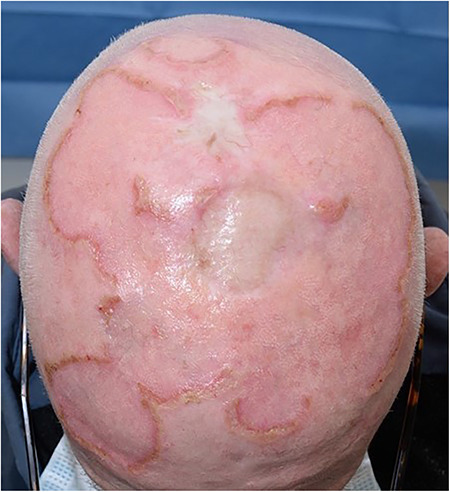

A 77‐year‐old man under review for non‐melanoma skin cancer in the context of a previous cardiac transplant, presented with an asymptomatic scalp eruption which had evolved over the preceding 4 weeks. Immunosuppressive medications included ciclosporin 90 mg twice daily (2.5 mg/kg/day) and mycophenolate mofetil 1 g twice daily.

Physical examination revealed well‐defined annular and polycyclic patches with brownish crusting at the peripheral margin distributed across his frontal and parietal scalp. A 4‐week course of clobetasol propionate 0.05% ointment was trialed empirically followed by a further 4 weeks course of fusidic acid 2% in combination with betamethasone valerate 0.1% cream but these were without benefit.

Samples were obtained for microbiological examination. Skin scrapings indicated no evidence of fungal infection. Bacterial culture demonstrated a heavy growth of *Staphylococcus aureus* sensitive to flucloxacillin. The patient was treated with 7 days of flucloxacillin 500 mg four times daily. Despite this, the eruption extended (Figure 1a).

**FIGURE 1:**
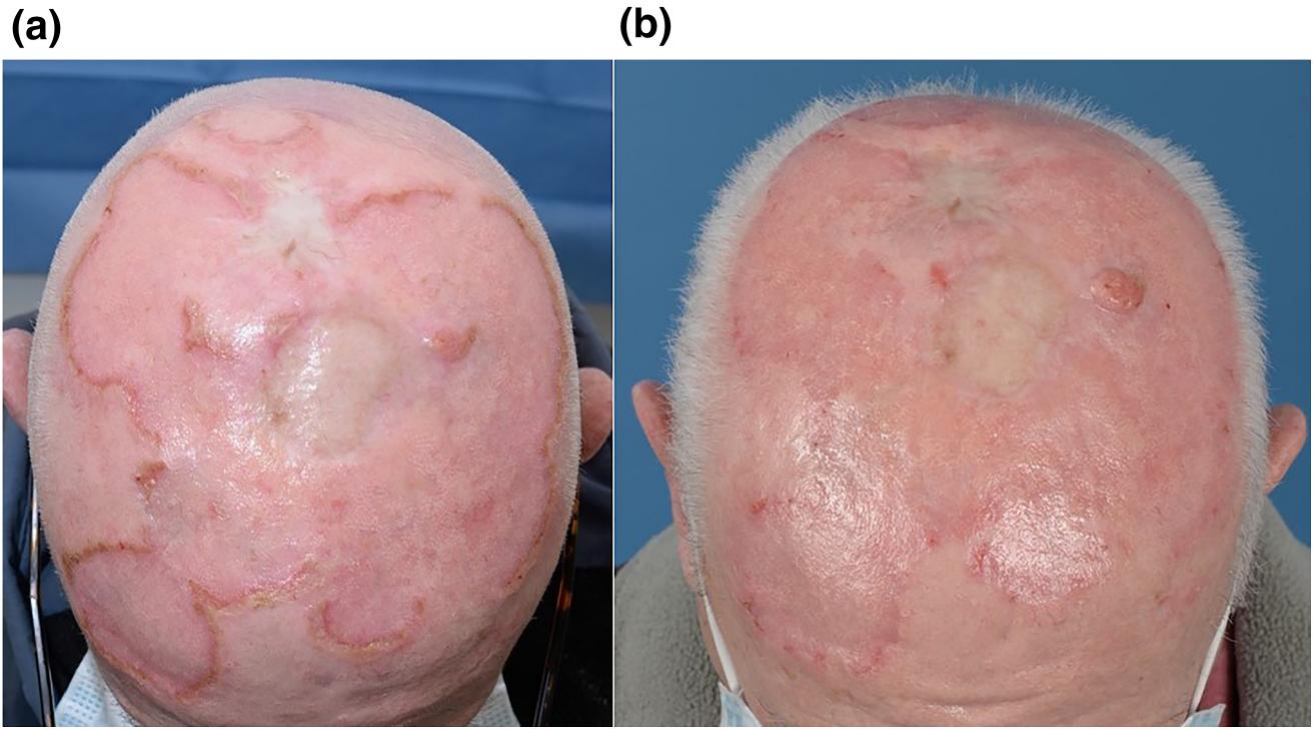
(a) Annular and polycyclic scalp eruption with peripheral crusting. (b) Clinical improvement following oral ciprofloxacin and 1% hydrogen peroxide cream. Incidental squamous cell carcinoma at left parietal scalp which has subsequently been excised.

An incisional skin biopsy was obtained from the scalp. Histopathology demonstrated epidermal spongiosis, florid dermal inflammatory cell infiltrate and abundant bacteria and neutrophils in the parakeratotic crust (Figure 2a). The bacteria were found to be gram‐negative on Gram staining (Figure 2b). Stains for fungus and acid‐fast bacilli were negative. Direct immunofluorescence was performed to exclude pemphigus foliaceus and was negative.

**FIGURE 2:**
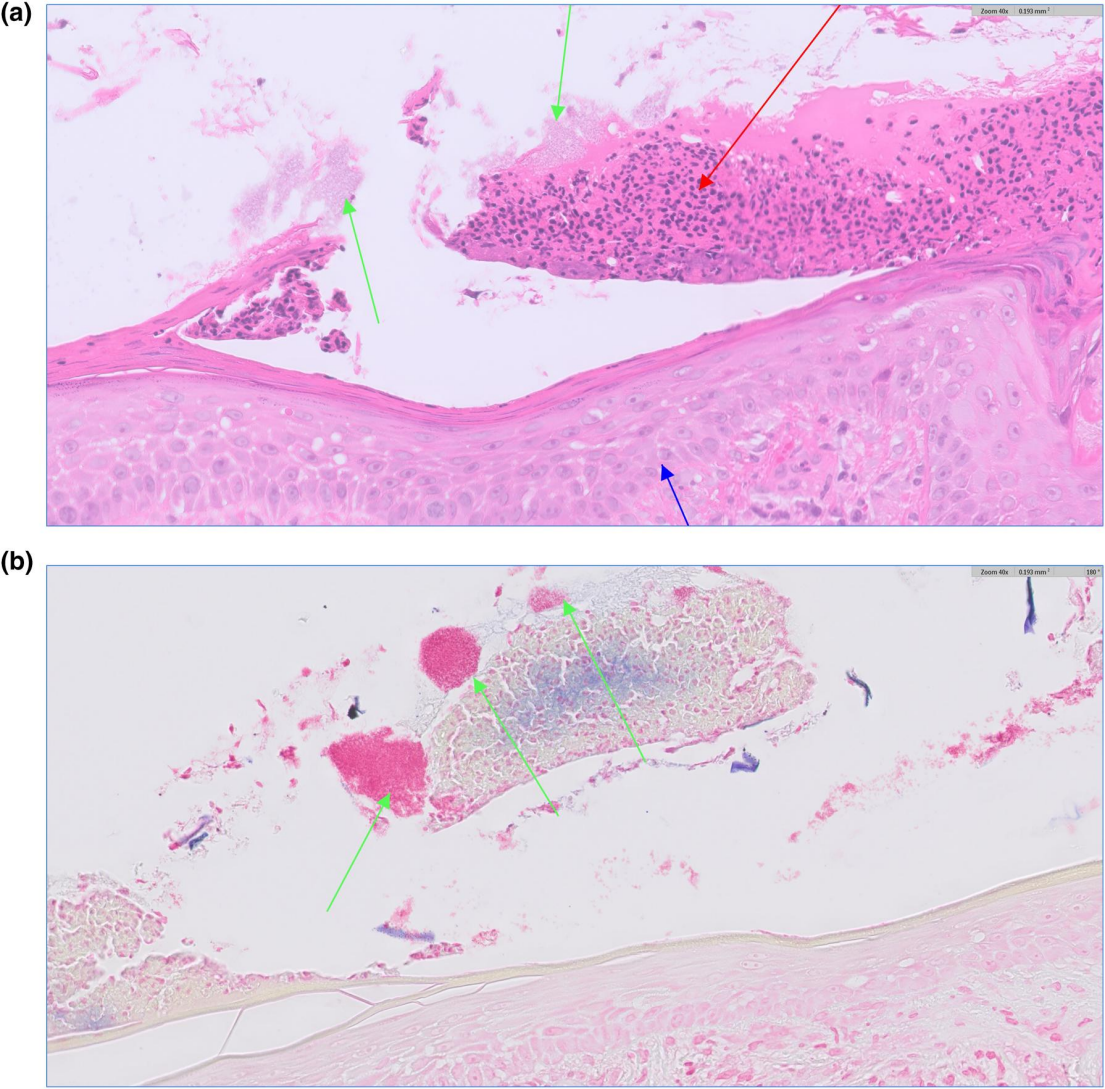
(a) Aggregates of bacteria (green arrows) on the skin surface mixed with neutrophils (red arrow). Keratinocytes of the epidermis (blue arrow) for orientation. Haematoxylin and eosin, original magnification ×400. (b) Same area as seen in panel (a) stained with Gram stain. Gram staining shows that the aggregates of bacteria (green arrows) are Gram negative as they are staining pink. It is not possible to comment further on morphology on maximum magnification. Original magnification ×400.

A repeat sample was obtained for bacterial culture. This demonstrated a heavy growth of *Serratia marcescens* sensitive to ciprofloxacin. The patient was treated with a 10‐day course of oral ciprofloxacin 500 mg twice daily along with 1% hydrogen peroxide cream topically. After 2 weeks of treatment, there was significant clinical improvement (Figure 1b).

Previous microbiology samples were reviewed retrospectively. It was noted that a coliform bacillus was present in the initial scalp swab but had not been reported. The coliform had been thought to represent colonisation as the clinical history of immunosuppression had not been provided with the sample. In addition, *S. marcescens* was also detected in a swab from a transient eruption on the chest 4 months earlier. A swab of this chest eruption 2 months prior was reported as no significant growth despite the presence of coliform which was disregarded, again due to lack of relevant history on the request form. Further investigation revealed that a heavy growth of a coliform bacillus was demonstrated in a similar eruption on the chest 9 years prior. It was concluded likely that the patient was chronically colonised with *S. marcescens* which had caused intermittent superficial skin infections over several years. All microbiology samples were obtained in primary care, the dermatology outpatient department or on first presentation to hospital suggesting community acquired infection.

*S. marcescens* is a facultative anaerobic gram‐negative bacillus and a member of the *Enterobacteriaceae* family.^1^ Community‐acquired infections are thought to account for around half of *Serratia* bacteraemia cases. Environmental sources include water, soil and animals.^2^ *S. marcescens* is commonly found within water and water‐pipes and is able to survive a variety of disinfectants, thereby also acting as an important cause of nosocomial infection as once the environment is colonised with this organism, it is difficult to eradicate.^1^ *S. marcescens* produces a distinctive red pigment, prodigiosin, which is often seen on bars of soap, shampoo bottles and on grout around sinks.^3^ Previous outbreaks of *S. marcescens* in hospital settings are well documented in the literature, usually as a result of the organism transfer via the hands of healthcare staff.^4^ There is also literature describing the contamination of premade syringes and giving‐sets.^5^

*Serratia* species secrete several virulence factors including an exotoxin (haemolysin Sh1A), a protease enzyme (serralysin) and formation of a biofilm.^2^ *S. marcescens* is inherently resistant to narrower spectrum β‐lactams and cephalosporins. Resistance to broader spectrum beta‐lactams is by way of chromosomal AmpC β‐lactamase enzyme, plasmid–encoded β‐lactamases, plasmid–mediated extended spectrum β‐lactamases and plasmid‐mediated carbapenemases.^6^ Resistance to other classes of antibiotics such as aminoglycosides and fluoroquinolones is attributed to a combination of mutations as well as chromosomally and plasmid mediated mechanisms.^6^

Reports of primary cutaneous infection as a result of *S. marcescens* are rare and typically occur in the context of immunosuppression.^1^, ^7^, ^8^ Skin infection can present either acutely with life‐threatening cellulitis, abscesses, ulcers and necrotising fasciitis, or more indolently as a chronic infection in the form of nodules or granulomatous lesions.^1^, ^7^ In a review of 10 cases of soft tissue infection caused by *S. marcescens*, the onset of symptoms was rapid and associated with systemic upset. Of the eight patients with reported outcomes, four died highlighting the potentially serious nature of *S. marcescens* infection.^2^ Our patient had a more indolent clinical course, despite his immunosuppressed state.

Recipients of solid organ transplantation are at significant risk of infection due to chronic immunosuppression. Organisms within the patient's environment are an important potential source of infection.^9^ On detailed questioning, the patient highlighted long‐term use of ketoconazole shampoo, using the same bottle about three times per week for approximately 1 year. Given the propensity of *S. marcescens* for damp environments and its strong tendency to environmental colonisation, it was postulated that items such as his shampoo may have been a reservoir for infection. To reduce any potential re‐exposure, the patient was given advice regarding appropriate infection preventative measures for his home environment including using smaller bottles of shampoo which are more frequently renewed.

Microbiology results are interpreted and reported by the laboratory in the context of the clinical information provided with the sample. The growth of a gram‐negative organism observed on the initial scalp swap was not reported as it was thought to represent colonisation rather than a pathogen. However, had the patient's immunosuppressed state been described in the clinical information, this may have led to more timely identification and treatment of the causative organism. Relevant information must be supplied with microbiology requests to allow accurate interpretation of results and consideration of organisms which may otherwise be overlooked or considered contaminants or colonisers.

This case highlights an unusual recurrent cutaneous clinical presentation of *S. marcescens* and the importance of considering this organism in the differential diagnosis of skin eruptions in immunocompromised patients. Furthermore, this case demonstrates the importance of providing detailed clinical information when performing diagnostic tests to allow accurate interpretation and reporting of results.

## CONFLICT OF INTEREST STATEMENT

None to declare.

## AUTHOR CONTRIBUTIONS

**Sarah E. Drummond**: Project administration (lead); writing—original draft (equal). **Akash Maliampurakal**: Writing—original draft (equal). **Saranaz Jamdar**: Writing—review & editing (supporting). **Lucy Melly**: Writing—review & editing (supporting). **Susan Holmes**: Writing—review & editing (lead).

## ETHICS STATEMENT

Not applicable.

## Data Availability

Data sharing not applicable—no new data generated.
